# The Effects of Peanuts and Tree Nuts on Lipid Profile in Type 2 Diabetic Patients: A Systematic Review and Meta-Analysis of Randomized, Controlled-Feeding Clinical Studies

**DOI:** 10.3389/fnut.2021.765571

**Published:** 2021-12-01

**Authors:** Jia-yue Xia, Jun-hui Yu, Deng-feng Xu, Chao Yang, Hui Xia, Gui-ju Sun

**Affiliations:** ^1^Key Laboratory of Environmental Medicine and Engineering of Ministry of Education, School of Public Health, Southeast University, Nanjing, China; ^2^Department of Nutrition and Food Hygiene, School of Public Health, Southeast University, Nanjing, China

**Keywords:** nuts, peanut, cholesterol, blood lipid, type 2 diabetes mellitus, meta-analysis

## Abstract

**Background:** Type 2 diabetes mellitus was found to be associated with metabolic disorders, particularly abnormal glucose and lipid metabolism. Dietary food choices may have profound effects on blood lipids. The primary objective of this study was to examine the effects of peanuts and tree nuts intake on lipid profile in patients with type 2 diabetes.

**Methods:** According to preferred reporting items for systematic reviews and meta-analysis guidelines, we performed a systematic search of randomized controlled clinical trials and systematic reviews published in PubMed, Web of Science, Embase, Scopus, and Cochrane library, from inception through June 2021. Studies in populations with type 2 diabetes, which compare nuts or peanuts to a controlled-diet group were included. We used the mean difference with 95% CIs to present estimates for continuous outcomes from individual studies. In addition, we used the GRADEpro tool to evaluate the overall quality of evidence.

**Results:** Sixteen studies involving 1,041 participants were eligible for this review. The results showed that peanuts and tree nuts supplementation did not induce significant changes in low-density lipoprotein-cholesterol (LDL-C) (mean difference = −0.11; 95%CI: −0.25 – 0.03, *p* = 0.117) and high-density lipoprotein-cholesterol (HDL-C) (mean difference = 0.01; 95%CI: −0.01 – 0.04, *p* = 0.400) in patients with type 2 diabetics. In addition, we found that peanuts and tree nuts intake may cause a significantly reduction in total cholesterol (TC) (mean difference = −0.14; 95%CI: −0.26 – −0.02, *p* = 0.024) and triglyceride (TG) (mean difference = −0.10; 95%CI: −0.17 – −0.02, *p* = 0.010). In the subgroup analysis, a significantly greater reduction in TC was observed in studies which duration was <12 weeks (mean difference = −0.22; 95%CI: −0.37 – −0.08, *p* = 0.002). The quality of the body of evidence was “moderate” for TC and TG, the quality of evidence for LDL-C and HDL-C were “low.”

**Conclusion:** Our findings suggest that consuming peanuts and tree nuts might be beneficial to lower TC concentration and TG concentration in type 2 diabetics subjects. Furthermore, peanuts and tree nuts supplementation could be considered as a part of a healthy lifestyle in the management of blood lipids in patients with type 2 diabetes. Given some limits observed in the current studies, more well-designed trials are still needed.

## Introduction

Type 2 diabetes mellitus (T2DM), as the most common progressive metabolic disease, is primarily characterized by insulin resistance, a relative deficiency of insulin, and high blood glucose in specific organs and organisms (1). In the United States, the number of patients with diabetes reached ~23.6 million, and only 17.9 million of them have been diagnosed according to the National Diabetes Statistics (2). T2DM is implicated in disorders of glucose and lipid metabolism (3). Patients with diabetes seem to primarily focus on blood glucose levels and may consider lipid control as less important (4, 5). Dyslipidemia in association with type 2 diabetes, which leads to a high rate of severe cardiovascular diseases, is the major cause of morbidity and mortality (6). Thus, intensive management of lipid concentrations to lower the risk of complications of disease progression is imperative.

Nuts (mainly refer to tree nuts and peanuts), belong to high-density nutritious foods, are rich in beneficial nutrients such as unsaturated fatty acids, fiber, lignans, vitamins, minerals, and other biologically active ingredients (7). The most common and popular edible nuts worldwide include walnuts, almonds, hazelnuts, and pistachios. In addition, it also included macadamias, pine nuts, cashews, pecans, and Brazil nuts. Definition of nuts, from a consumer perspective, also contains peanuts which belong to peanuts or beans in botanical terms, but are largely included in the nut food group. In addition, the nutritional content of peanuts is similar to that of tree nuts (8, 9). Nowadays tree nuts, integrated in the Mediterranean Diet Pyramid, are regarded as essential parts of a healthy diet (10).

Over the past years, a large body of evidence has accumulated regarding the relationship between peanuts and tree nuts consumption and health outcomes from both epidemiological and controlled dietary trials (11–13). Studies from epidemiology certainly indicated that mature persons more than 65 years old, supplementing three or more servings of nuts every week, had a lower risk of obesity, diabetes, and metabolic syndrome (14). A meta-analysis included 13 clinical trials revealed that the walnut diets can significantly reduce the low-density lipoprotein-cholesterol (LDL-C) concentration compared with the control diet. Walnut diets or control diets could not induce significant changes in high-density lipoprotein-cholesterol (HDL-C) and triglycerides (TGs) (15). However, a meta-analysis in 2015 (16) revealed that supplementation of tree nuts may have a significant reduction effect on total cholesterol (TC), LDL-C, and TGs concentration. In addition, Mejia et al. (17) concluded that tree nuts could benefit the metabolic syndrome through a modest decrease in TGs. The conclusions of these studies or reviews, obviously, remain controversial. Although the effects of different types of nuts have been studied in different populations such as hyperlipidemic/hypercholesterolemic population (18, 19), healthy subjects (20, 21), and population with type 2 diabetes (22–24), and some systematic review and meta-analysis have been conducted to explore the effects of different types of nuts consumption on lipid level, there have been relatively few meta-analyses that have specifically investigated the effects of peanuts and total or different types of tree nuts on lipid profile in the diabetic population, especially in patients with T2DM. Therefore, it is necessary to analyze and determine the effect of peanuts and tree nuts consumption on blood lipids in subjects with type 2 diabetes, and this is the novelty of this study.

To provide better evidence-based guidance on the role of peanuts and tree nuts on lipid profile in type 2 diabetic subjects, we conducted a systematic review and meta-analysis of randomized controlled-feeding dietary clinical trials to assess the effects of peanuts and tree nuts (include walnuts, pistachios, macadamia nuts, pecans, cashews, almonds, hazelnuts, pine nuts, and Brazil nuts) on lipid profile (LDL-C, HDL-C, TC, and TG) in individuals with type 2 diabetes.

## Materials and Methods

### Literature Search Strategy

We designed and conducted the study according to the guidelines of the 2020 preferred reporting items for systematic reviews and meta-analysis (PRISMA) statement (25). The search was performed in PubMed, Web of Science, Embase, Scopus, and Cochrane library without language restrictions for relevant literature from inception to June 30, 2021. The search used the terms (“Nuts” OR “Tree nuts” OR “pistachios” OR “pine nuts” OR “brazil nuts” OR “cashews” OR “hazelnuts” OR “almonds” OR “walnuts” OR “pecans” OR “macadamia nuts” OR “peanuts”) and (“Cholesterol, HDL” OR “Cholesterol, LDL” OR “Triglycerides” OR “serum lipids” OR “triglyceride” OR “low density lipoprotein” OR “high density lipoprotein” OR “TG” OR “TC” OR “lipid profile” OR “serum lipid”) and (“Type 2 diabetes” OR “diabetes mellitus, type II” OR “type 2 diabetes mellitus” OR “type 2 diabetes” OR “Diabetes, Type 2”) and (“randomized controlled trial” OR “randomized controlled trial” OR “controlled clinical trial” OR “clinical trial, randomized” OR “randomized, trial” OR “randomized” OR “intervention” OR “controlled trial” OR “random” OR “placebo”). If the references of the included kinds of literature meet the requirements, they will be further screened to identify potentially relevant studies. Two reviewers (JYX and JHY) independently conducted the data extraction, and any disagreements in terms of literature research were resolved through rigorous discussion or professional consultation. The details of the search strategy are shown in Supplementary Data 2.

### Study Selection

Original studies that met the following inclusion criteria were selected in the systematic reviews and meta-analysis: (a) randomized controlled trials which compare nuts or peanuts to a controlled-diet group; (b) the study population was limited only to the patients diagnosed with T2DM. Accordingly, the following exclusion criteria were also used: (a) studies that clearly did not fulfill the aforementioned inclusion criteria; (b) animal experiments, case reports, editorials, conference papers, and reviews.

### Data Extraction

Independent extraction of data from eligible articles was performed by two authors according to a specially designed data collection form. Data collection items covered the name of the first author, year of publication, country, mean participant age (year), and the number of subjects: intervention group (*n*) and control group (*n*), feeding control, study design, nut type, nuts dose (g/d), and follow-up period. Furthermore, SD and mean net change of LDL-cholesterol (mmol/l), HDL-cholesterol (mmol/l), TC (mmol/l), and TG (mmol/l) in each study were extracted.

### Quality Assessment

Literature quality evaluation of included studies was performed according to the Cochrane collaboration risk-of-bias tool (26), and the assessment tool assesses seven domains: random sequence generation, allocation concealment, blinding of participants and personnel, blinding of outcome assessment, incomplete outcome data, selective outcome reporting, and other bias. Based on the recommendations of the Cochrane Handbook, each item was scored as “high risk of bias,” “low risk of bias,” or “unclear risk” for all selected studies. Any disagreements between the authors were resolved through rigorous discussion.

### Quantitative Data Synthesis

Statistical analyses were conducted using Stata SE 15.0 (Stata Corporation, TX, USA) and RStudio v 4.0.3 (RStudio Inc., MA, USA). The package “meta” (version 4.19-0), and “ggplot2” (version 3.3.5) in RStudio were used in combination with Stata SE to perform all analyses. Original studies did not provide effective data directly, the mean net change of relevant indexes was calculated by subtracting mean change (end value minus baseline value). The last end value was used if there were many endpoints. For parallel-controlled studies, we calculated the mean difference (MD) in the effect by using the change from baseline in the control group and the change from baseline in the intervention group. For cross-over trials, the blood lipid value at the end of the control phase and the blood lipid value at the end of the intervention phase are extracted to compute the MD in the effect. The net change of SD was calculated based on the formula if studies did not provide the net change of SD. The formula was used as follows: SD_net change_=$\sqrt{SD{\left(baseline\right)}^{2}+\;SD{\left(end\;point\right)}^{2}-2R\times SD(baseline)\times \;SD(end\;point)\;}$. Where necessary, a correlation coefficient *R* was used and we calculated it according to the following formula from the Cochrane handbook: R = $\frac{\left[(SD{\left(baseline\right)}^{2}+SD{\left(end\;point\right)}^{2}-SD{\left(change\right)}^{2})\right]}{\left[2^{*}SD(baeline)*SD(end\;point)\right]}$. We assumed a correlation coefficient *R* = 0.5 according to Higgins et al. (27) and used it to calculate the SD of difference in crossover trials when the study did not provide or have sufficient data to calculate the correlation coefficient. We included trials in which tree nuts and peanuts were directly supplemented or added to the diet. Also, a weighted effect size was used for trials involving multiple interventions in a single study. Paired analyses were performed for all multiple intervention studies (28). We had converted the units from mg/dl to mmol/l for the meta-analysis. For TC, LDL-C, and HDL-C, 1 mg/dl was converted to 0.0259 mmol/l; for TG, 1 mg/dl was converted to 0.0113 mmol/l. Statistical significance was defined as two-sided *p* < 0.05. The extraction of data from the included studies was combined using the generic inverse variance method and a fixed- and random-effects model depending on the degree of heterogeneity. Heterogeneity was assessed through the *I*^2^ metrics and chi-squared statistics, either *I*^2^ > 50% or *p*-value of χ^2^ test < 0.10 was deemed as statistically significant heterogeneity. We used the MD with 95% CIs to present estimates for continuous outcomes from individual studies. Sources of heterogeneity were explored using sensitivity and subgroup analyses. To determine whether a single study has an undue influence on the overall results, sensitivity analyses were performed by excluding the studies sequentially and repeating the meta-analysis. Sensitivity analyses were also by using the random-effects model with Hartung–Knapp–Sidik–Jonkman adjustment or using correlation coefficient of 0.25, 0.5, and 0.75 for cross-over studies to ascertain whether the overall results were robust to the use of different derived correlation coefficients. Also, we conducted subgroup analyses by prespecified covariates, namely, nut type, nut dose (g/d), the mean age of participants, study design, duration of trials, controlled diets, and tested these covariates for significance by meta-regression.

### Publication Bias

We assessed the asymmetry of the funnel plot visually, used Begg's rank correlation test and Egger's linear regression test to explore any potential publication bias. Also, a “trim and fill” analysis was used to further observe the stability of results if there was any asymmetry in the funnel plot (29).

### Certainty of Evidence

We used the GRADEpro Guideline Development Tool (www.gradepro.org) to rate the quality of evidence and strength of recommendations for each outcome. Quality of a body of evidence involves consideration of the within-study risk of bias (methodological quality), directness of evidence, heterogeneity, the precision of effect estimates, and risk of publication bias. The GRADE system offers four categories of the quality of the evidence (high; moderate; low; and very low) (30).

## Results

### Study Identification and Selection

**Figure 1:**
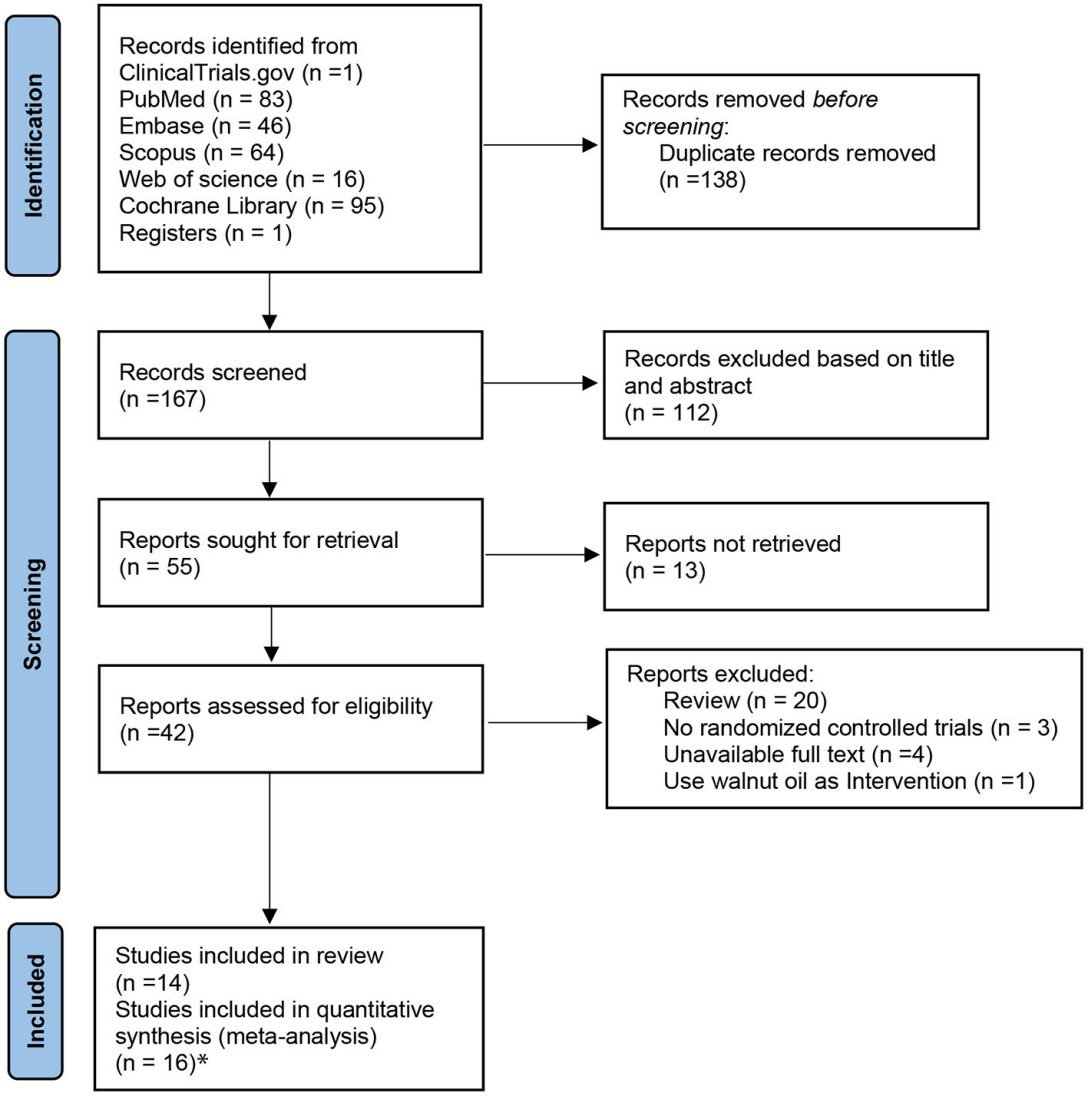
The PRISMA flow diagram of selected trials. Asterisk represents that studies by Jenkins et al. and Lovejoy et al. were each divided into two trials. PRISMA, preferred reporting items for systematic reviews and meta-analysis.

A PRISMA flow diagram of the included trials is shown in Figure 1. A total of 305 articles were identified with the preset search strategy from five electronic databases and one website (ClinicalTrials.gov), of which 250 articles were removed based on duplicate articles, titles, and abstracts. Then, 55 full-text trials were retrieved and only 42 trials were included to be assessed for eligibility; of those, 28 studies were excluded for the following reasons: review articles (*n* = 20), full articles are unavailable (*n* = 4), non-randomized controlled trials (*n* = 3), and using walnut oil as intervention (*n* = 1). Finally, two of the 14 included studies were divided into two trials, respectively. The study by Lovejoy et al. was divided since it included high almond with high fat and high almond with low fat (31). Jenkins administered two doses of nuts, namely, full-nut dose (mean intake 73 g/day) and half-nut dose (32). Therefore, 16 trials with sufficient data were eligible for inclusion in the final analysis.

### Study Characteristics

The main characteristics of the included studies are summarized in Table 1 and Supplementary Data 3. These studies were published between 2002 and 2019. Four of them were performed in Asian countries [Taiwan (China) (33, 34) and Iran (22, 35)], and the other 12 studies were from the United States (31, 36–40), Canada (41), Australia (42, 43), and India (44). Nine studies (22, 35, 38, 39, 41–44) have a parallel design, while seven (31, 33, 34, 36, 37, 45) have a cross-over design. The sample size ranged from 19 to 300 for a total number of 1,041. The mean age of participants was between 50.1 and 66 years of age. The follow-up period of included studies ranges from 2 weeks to 12 months.

**Table 1:** Characteristics of included 16 trials in the systematic review and meta-analysis (mean values and standard deviations).

**References**	**Year**	**No. of patients**	**Study region**	**Mean age, y (SD)**	**Female (%)**	Comparison group^*^	**Study design**	**Nut type**	**Nut dose, g/d**	**Follow-up**
Lovejoy et al.-HF (31)	2002	30	USA	53.8 (10.4)	17 (50%)	High-fat control (37% total fat)	Crossover	Almond	57–113 g	6w
Lovejoy et al.-LF (31)	2002	30	USA	53.8 (10.4)	17 (50%)	Low-fat control (25% total fat)	Crossover	Almond	57–113 g	6w
Tapsell et al. (42)	2004	58	Australia	59.3 (8.1)	18 (31%)	Low-fat control (low fat, <30% fat)Parallel	Walnuts	30 g	6m
Tapsell et al. (43)	2009	50	Australia	54 (8.7)	unclear	Normal fat ratio control (30% fat, 20% protein, 50% carbohydrate)	Parallel	Walnuts	30 g	12m
Ma et al. (36)	2010	24	USA	58.1 (9.2)	14 (58%)	Self-selected casual diet	Crossover	Walnuts	56 g	8w
Li et al. (33)	2011	22	Taiwan (China)	58 (2)	11 (55%)	Low-fat control (27% fat, 17% protein, 56% carbohydrate)	Crossover	Almonds	56 g	12w
Jenkins et al.-FD (32)	2018	117	Canada	62 (9.4)	27 (23%)	Low-fat control (Muffins, total fat <50 g)	Parallel	Mixed nuts	75 g	3m
Jenkins et al.-HD (32)	2018	117	Canada	61 (8.9)	25 (21%)	Low-fat control (Muffins, total fat <50 g)	Parallel	Mixed nuts	37.5 g	3m
Cohen et al. (37)	2011	19	USA	66 (3.3)	14 (74%)	Low-fat control (Cheese, 12 g fat)	Crossover	Almonds	29 g	12w
Damavandi et al. (35)	2013	50	Iran	55.7 (7.7)	34 (68%)	Self-selected casual diet	Parallel	Hazelnuts	29 g	8w
Sweazea et al. (38)	2014	24	USA	56.2 (7.4)	12 (57%)	Diabetic diet	Parallel	Almond	6–9 g	12w
Wien et al. (39)	2014	60	USA	61.5 (12.6)	30 (50%)	an ADA meal plan (35% total fat, 45% carbohydrate, 20% protein) and tree nuts	Parallel	Peanuts	2 oz per day (56.7 g)	24w
Sauder et al. (40)	2015	30	USA	56.1 (7.8)	15 (50%)	Low-fat control (26.9% total fat, 186 mg/day cholesterol)	Crossover	Pistachios	59–128 g	2w
Chiao-Ming et al. (34)	2017	40	Taiwan (China)	54.9 (10.5)	20 (61%)	Low-fat control (28% fat, 17% protein, 55% carbohydrate)	Crossover	Almonds	60 g	28w
Mohan et al. (44)	2018	300	India	50.8 (9.5)	15 (52%)	Diabetic diet	Parallel	Cashew	30 g	12w
Sedaghat et al. (22)	2019	70	Iran	50.1 (8.7)	38 (586%)	Diabetic diet	Parallel	Soy nut	60 g	8w

*HF, high fat; LF, low fat; FD, full dose; HD, half dose.*

* *We define a daily diet with a fat ratio >30% or an intake >50 g as a high-fat control diet, and vice versa is a low-fat control diet*.

### Risk of Bias Assessment

**Figure 2:**
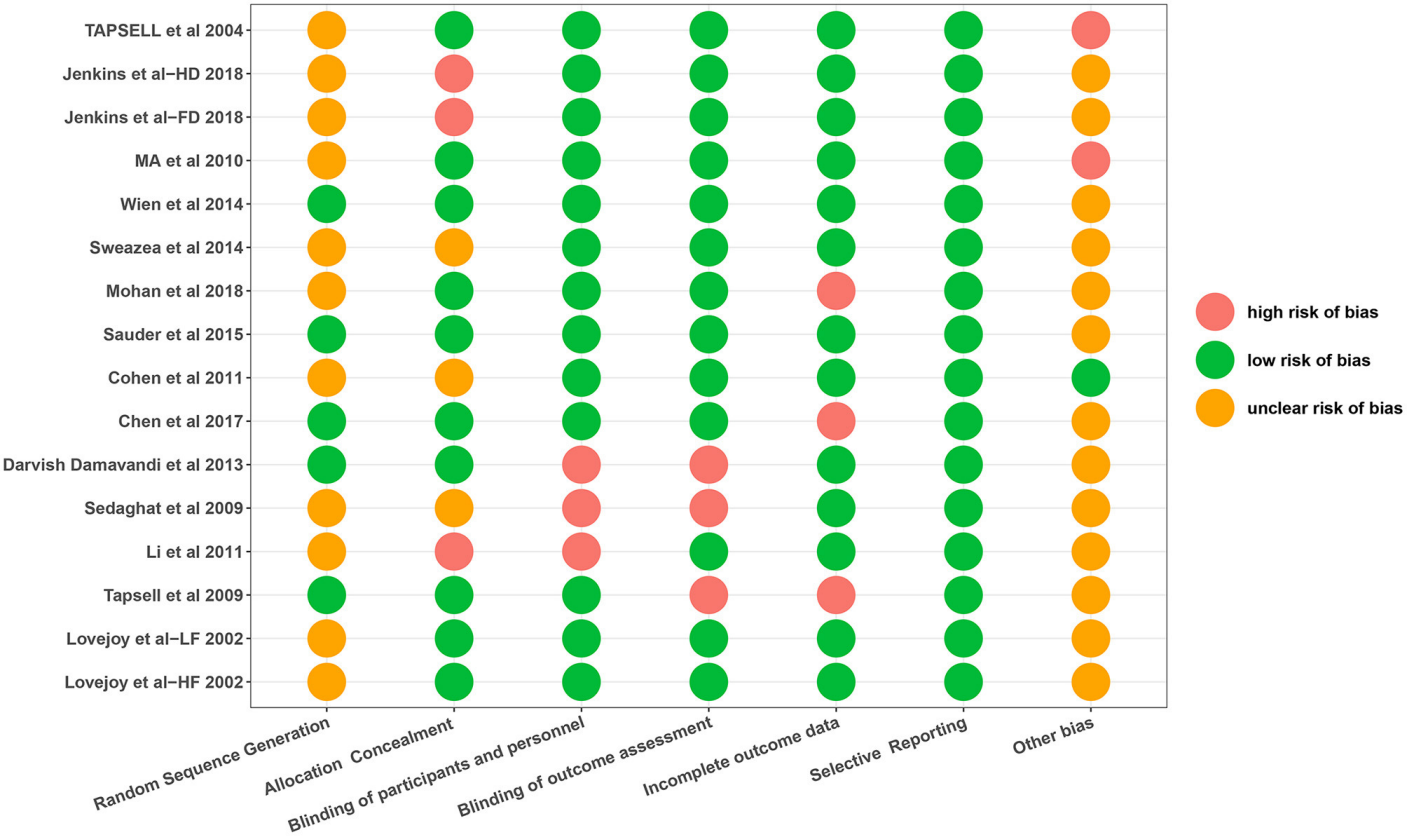
Risk of bias assessment.

The risk of bias assessments is shown in Figure 2. Most studies were rated as at low risk of bias. In addition, the risk of random sequence generation in the majority of included trials was unclear because there were insufficient information to generate a risk rating.

### Effect of Peanuts and Tree Nuts on LDL-C

**Figure 3:**
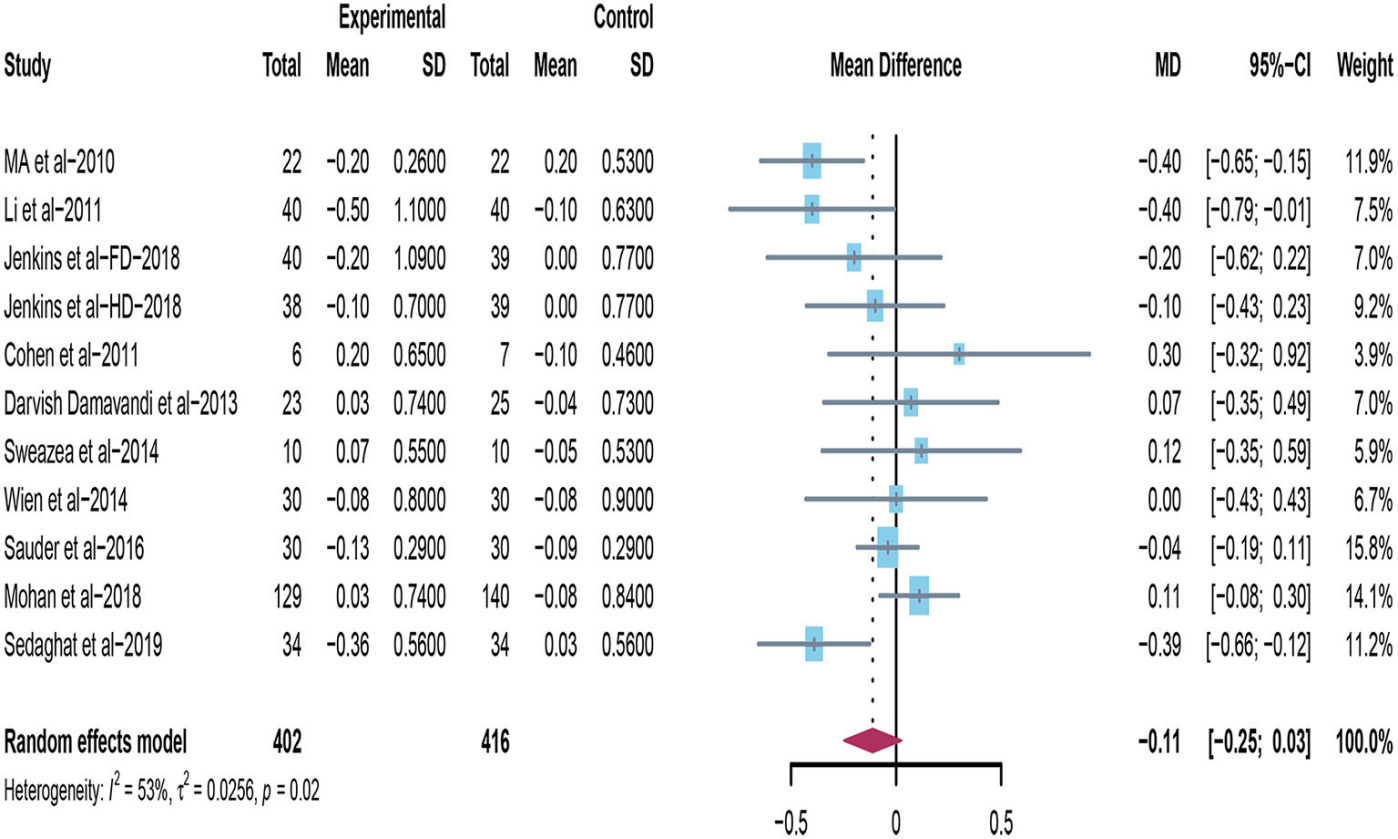
Effect of peanuts and tree nuts supplementation on LDL-C (mmol/l) in patients with type 2 diabetes. LDL-C, low-density lipoprotein-cholesterol.

Eleven trials conducted intervention dietary trials on estimating the effects of peanuts and tree nuts consumption on LDL-C in patients with type 2 diabetes (22, 32, 33, 35–40, 44). Four studies (22, 32, 33, 36) found that tree nuts or peanuts intake significantly decreased LDL-C. However, when 11 studies were included in the meta-analysis (*n* = 818), the result revealed that consuming tree nuts or peanuts did not cause a significant reduction in LDL-C in patients with T2DM (mean difference = −0.11; 95%CI: −0.25 – 0.03, *p* = 0.117) (Figure 3). There was significant heterogeneity exhibited between studies (*I*^2^ = 53%, *p* = 0.02).

### Effect of Peanuts and Tree Nuts on HDL-C

**Figure 4:**
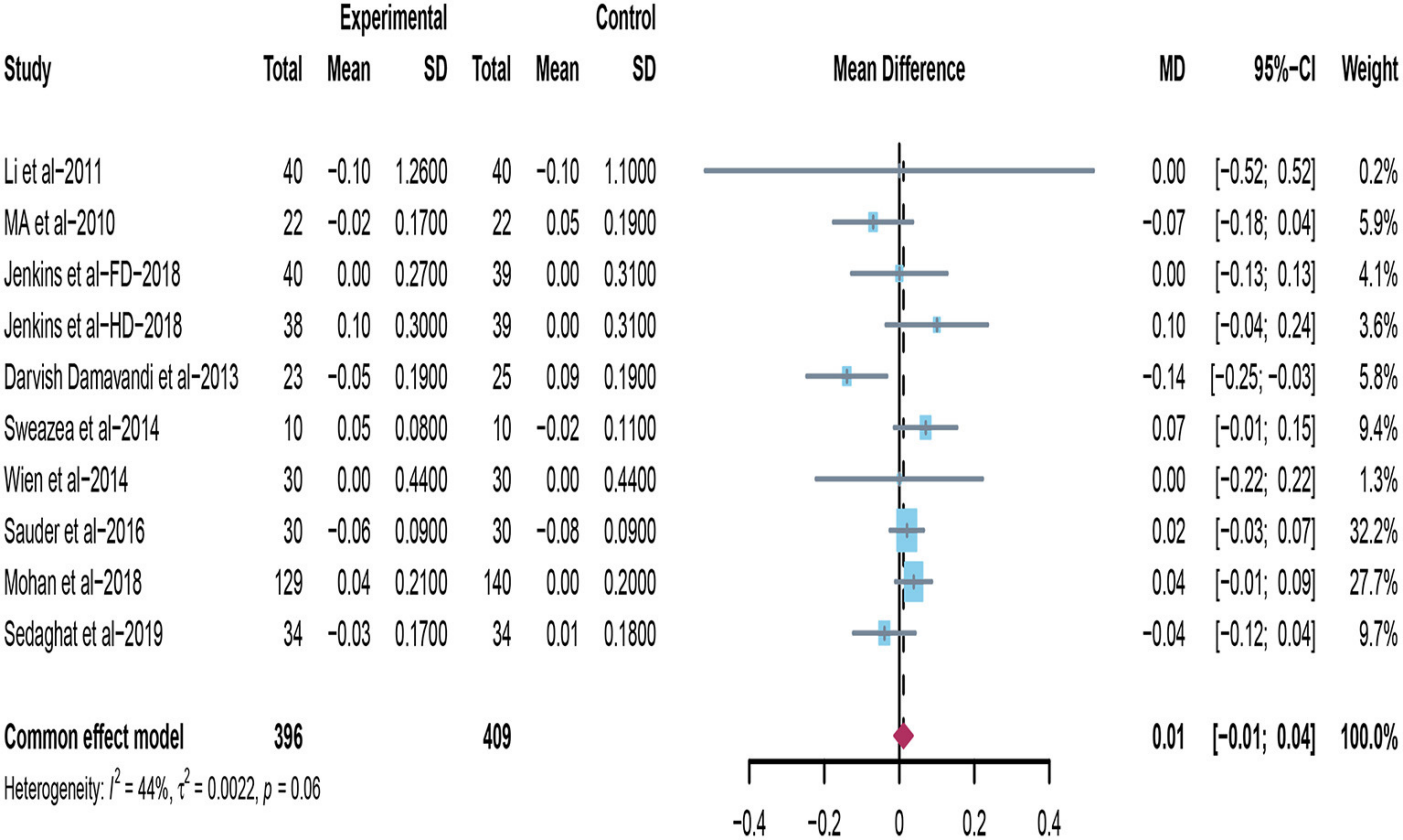
Effect of peanuts and tree nuts supplementation on HDL-C (mmol/l) in patients with type 2 diabetes. HDL-C, high-density lipoprotein-cholesterol.

Ten studies investigated the effects of supplementing peanuts and tree nuts on HDL-C in patients with type 2 diabetes (22, 32, 33, 35, 36, 38–40, 44). Damavandi et al. (35) found that the tree nuts group significantly decreased HDL-C. Mohan et al. (44) found that participants in the nuts group have a greater increase in HDL cholesterol compared with controls. However, when the data from included trials were pooled for meta-analysis (*n* = 805), it revealed that consuming tree nuts or peanuts have no significant reduction on HDL-C in patients with T2DM (mean difference = 0.01; 95%CI: −0.01 – 0.04, *p* = 0.400) (Figure 4), and no significant between-studies heterogeneity was observed (*I*^2^ = 44%, *p* = 0.06).

### Effect of Peanuts and Tree Nuts on TC

**Figure 5:**
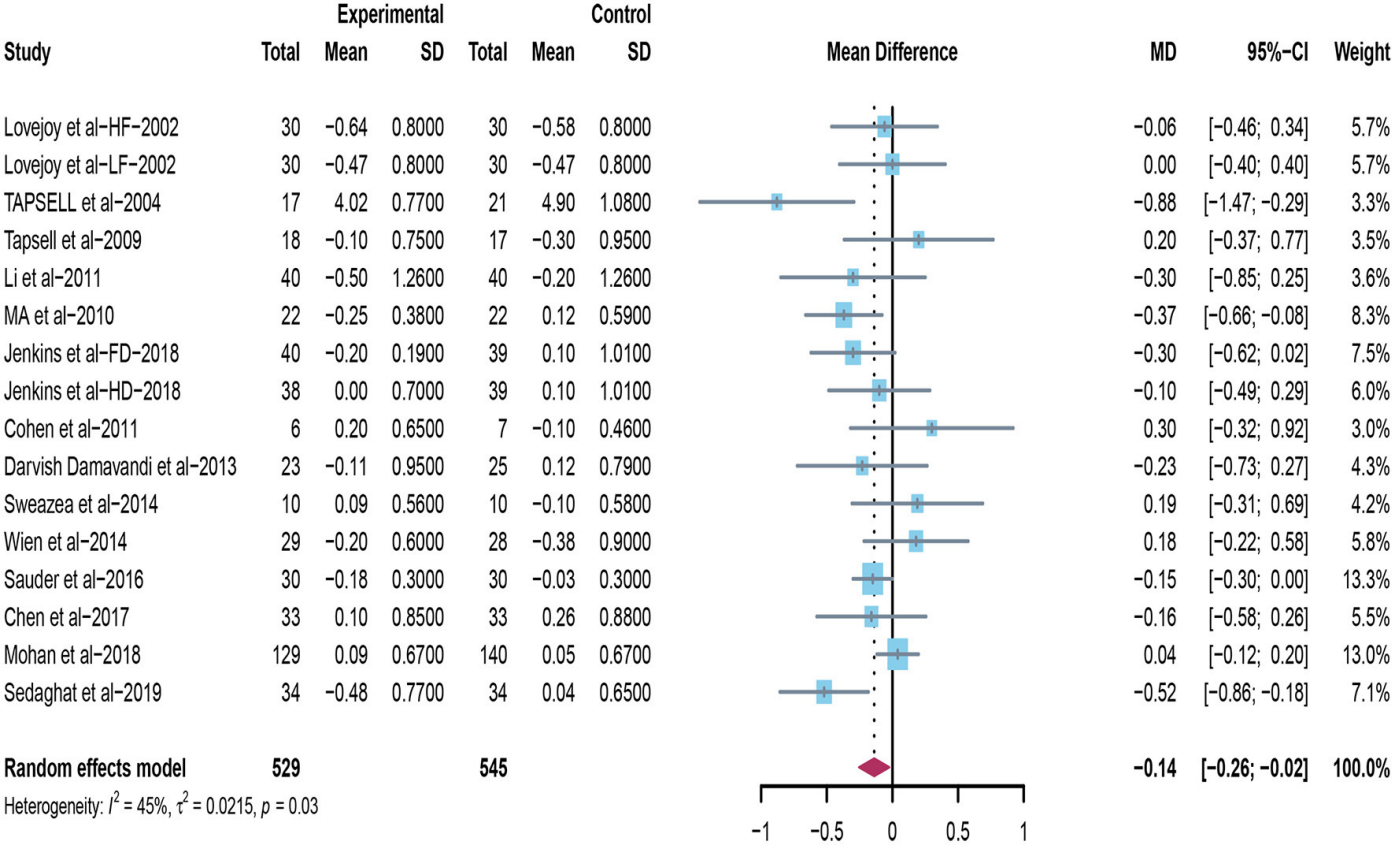
Effect of peanuts and tree nuts supplementation on total cholesterol (mmol/l) in patients with type 2 diabetes.

Sixteen trials explored the effects of peanuts and tree nuts supplementation on TC (22, 31–40, 42–44). Only three studies (22, 40, 42) found that tree nuts and peanuts significantly decreased TC. A meta-analysis included 16 studies (*n* = 1,074) indicated that peanuts and tree nuts consumption can significantly reduce TC concentration in patients with T2DM (mean difference = −0.14; 95%CI: −0.26 – −0.02, *P* = 0.024) (Figure 5). We observed a significant between-studies heterogeneity (*I*^2^ = 45%, *p* = 0.03).

### Effect of Peanuts and Tree Nuts on TG

**Figure 6:**
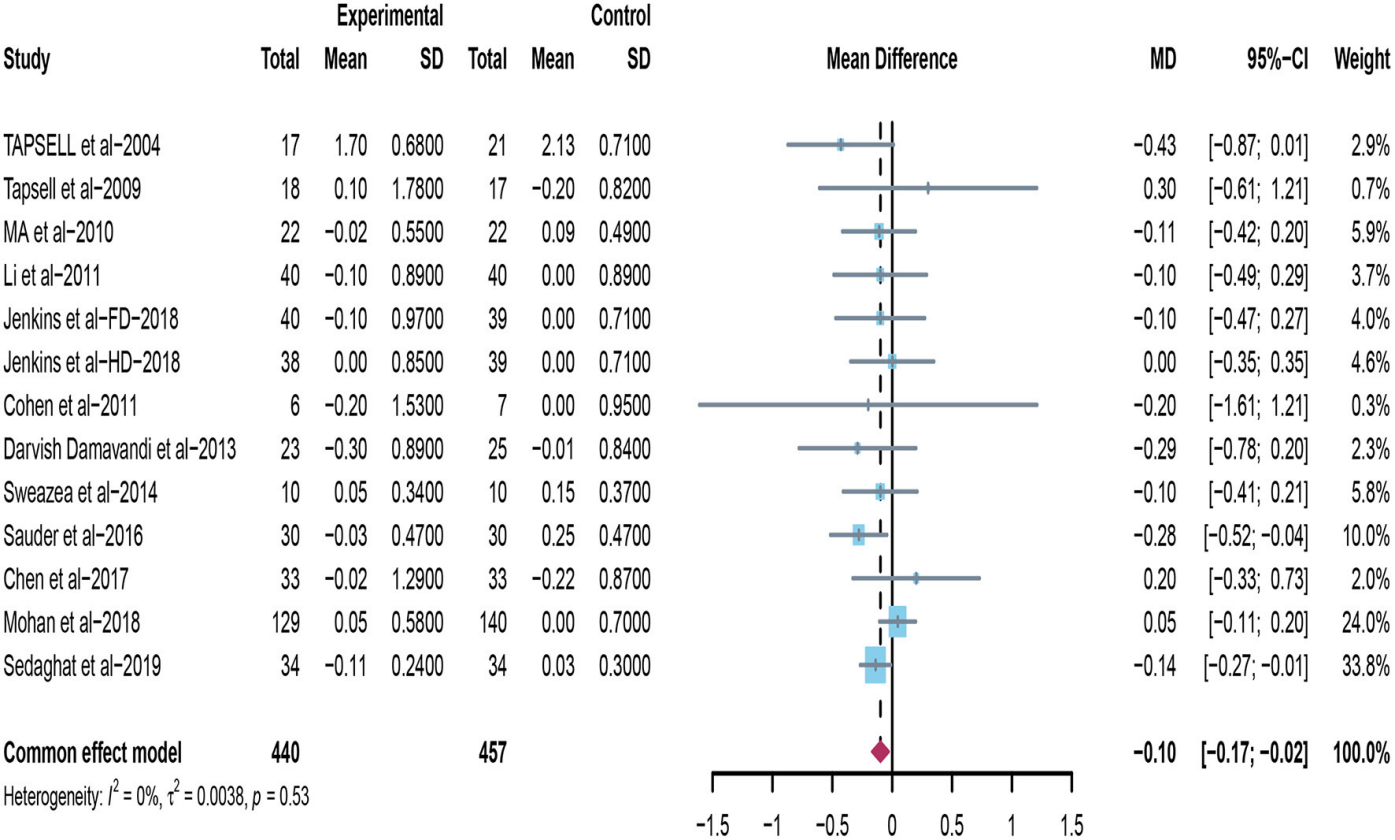
Effect of peanuts and tree nuts supplementation on triglyceride (mmol/l) in patients with type 2 diabetes.

Thirteen studies appraised the effects of peanuts and tree nuts on TG (22, 32–38, 40, 42–44). Only two trials (22, 40) found that the tree nuts group significantly decreased TG. Moreover, when the data from included trials (*n* = 897) was pooled for meta-analysis, it revealed that peanuts and tree nuts intake can significantly lower TG concentration in patients with T2DM (mean difference = −0.10; 95%CI: −0.17 – −0.02, *p* = 0.010) (Figure 6), and we found no significant heterogeneity between studies (*I*^2^ = 0%, *p* = 0.53).

### Subgroup Analysis

The effects of peanuts and tree nuts intake on LDL-C and TC in subgroups based on characteristics of included studies are presented in Supplementary Data 7. For TC, the results suggested that the duration of trials might be the heterogeneity source, and a significantly greater reduction in TC concentration was observed in studies whose duration was <12 weeks. However, we did not observe significant differences in effects by nut type, given that relatively few trials of certain nut types were available. For LDL-C, we did not find the sources of significant heterogeneity in the subgroup analysis. Furthermore, we conducted the meta-regression but did not find the relevant sources of significant heterogeneity.

### Sensitivity Analysis

We performed a sensitivity analysis to evaluate the stability of our present results. The overall results generally were robust to the use of different derived correlation coefficients (Supplementary Data 4). Also, no significant changes were observed when we used the random-effects model with Hartung-Knapp–Sidik–Jonkman adjustment (Supplementary Data 5). In addition, sensitivity analysis showed that the removal of any of the studies from our study did not considerably change the effect of peanuts and tree nuts consumption on LDL-C, HDL-C, TC, and TG (Supplementary Data 6). These results further suggest that our results are reliable and robust.

### Publication Bias Regarding Peanuts and Tree Nuts' Effect on Lipid Profile

**Figure 7:**
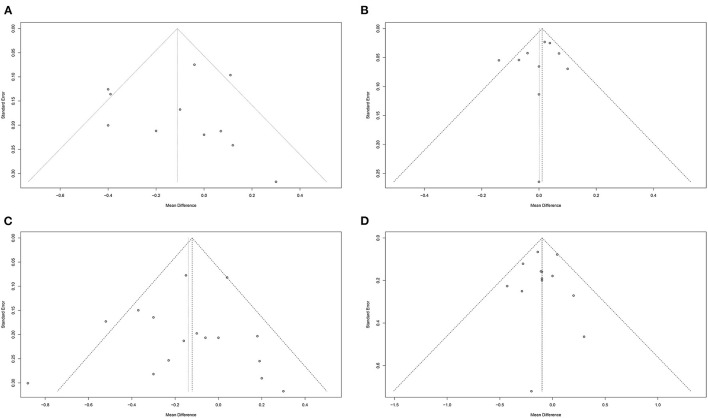
Funnel plots to evaluate publication bias, and effect of peanuts and tree nuts intake for **(A)** LDL-C Egger's test (*p* = 0.851), **(B)** HDL-C Egger's test (*p* = 0.460), **(C)** TC Egger's test (*p* = 0.670), and **(D)** TG Egger's test (*p* = 0.884) in patients with type 2 diabetes. HDL-C, high-density lipoprotein-cholesterol; LDL-C, low-density lipoprotein-cholesterol; TC, total cholesterol; TG, triglyceride; WMD, weighted mean difference.

We assessed potential publication biases by using Funnel plots, Begg's rank correlation test, Egger's regression test. Visual scanning of the funnel plot of LDL-C, HDL-C, TC, and TG suggested no asymmetry. These observations were confirmed by LDL-C Egger's regression test (intercept = −0.22; standard error = 1.12; 95% CI: −2.76 – 2.32; *t* = −0.19; two-tailed *p*-value = 0.851), HDL-C Egger's regression test (intercept = −0.68; standard error = 0.88; 95% CI: −2.74 – 1.34; *t* = −0.78; two-tailed *p*-value = 0.460), TC Egger's regression test (intercept = −0.34; standard error = 0.77; 95% CI: −1.99 – 1.32; *t* = −0.44; two-tailed *p*-value = 0.670), and TG Egger's regression test (intercept = −0.08; standard error = 0.53; 95% CI: −1.25 – 1.09; *t* = −0.15; two-tailed *p*-value = 0.884). Meanwhile, results did not indicate evidence of publication bias, which were confirmed by LDL-C Begg's rank correlation test (*z* with continuity corrected = 1.25; two-tailed *p*-value with continuity corrected = 0.213), HDL-C Begg's rank correlation test (*z* with continuity corrected = 0; two-tailed *p*-value with continuity corrected = 1.000), TC Begg's rank correlation test (*z* with continuity corrected = 0.27; two-tailed *P*-value with continuity corrected = 0.787), and TG Begg's rank correlation test (*z* with continuity corrected = 0.67; two-tailed *p*-value with continuity corrected = 0.502) (Figure 7).

### The Quality of the Body of Evidence

The quality of evidence was determined using the GRADE system (Supplementary Data 8). The quality of evidence was “moderate” for TC and TG, after being downgraded due to inconsistency. The quality of evidence for HDL-C was “low,” as a result of being downgraded for both inconsistency and imprecision. The quality of the body of evidence for LDL-C was “low,” as a result of being downgraded for both risk of bias and inconsistency.

## Discussion

To the best of our knowledge, dyslipidemia is associated with the development of type 2 diabetes, and few meta-analyses were found to comprehensively analyze pieces of evidence from randomized controlled trials on the efficacy of peanuts and tree nuts on lipids profile in patients diagnosed with T2DM. The novelty of our study is to explore the effects of peanuts and tree nuts consumption on lipid profile in type 2 diabetes population based on a certain amount of randomized controlled-feeding dietary trials. The results of our meta-analysis did not reveal any significant changes in LDL-C and HDL-C following supplementation with peanuts and tree nuts in patients with type 2 diabetes. Meanwhile, our results indicated a significant reduction of TC concentration (mean difference = −0.14; 95%CI: −0.26 – −0.02, *p* = 0.024) and TG concentration (mean difference = −0.10; 95%CI: −0.17 – −0.02, *p* = 0.010) following consuming peanuts and tree nuts in patients with T2DM. For TC, subgroup analysis revealed that the duration of trials might contribute to the heterogeneity source, and we found a significantly greater reduction in TC concentration among studies whose duration was <12 weeks. However, we did not observe significant differences in effects when the type of nut is limited. Furthermore, we did not find the sources of significant heterogeneity in the subgroup analysis for LDL-C. Obvious heterogeneity still existed in some subgroups, and we failed to explain the heterogeneity completely. In sensitivity analysis, the pooled estimates were relatively robust for LDL-C, HDL-C, TC, and TG.

Most outcomes concluded from our meta-analysis were similar to previous relevant studies. Blanco Mejia et al. (17) reported a significant decrease of TGs through consuming tree nuts in the overall analysis. A TG-reducing effect had also been observed in individual trials and meta-analysis of trials appraising the benefit of the Mediterranean diet in patients with T2DM (46). It was reported that the TG-decreasing effect was followed by an increased HDL-C (46). However, the present results revealed that peanuts and tree nuts did not significantly increase HDL-C concentration in patients with T2DM. It has been well-known that tree nuts contain many compounds such as polyunsaturated fatty acid, monounsaturated fatty acid, and fiber. To some extent, the benefits of tree nuts for improving blood lipid might be attributed to various kinds of nutrients which contribute to cardiovascular protection, such as unsaturated fatty acids, fiber, and phytochemicals (32). The fiber content and high unsaturated fat from tree nuts can substitute high glycemic index carbohydrates from the diet, thereby causing a reduction of the glycemic load in the diet, which may be the main facilitating factor in lowering TG (47).

Del Gobbo et al. (16) reported a significant decrease in TC and LDL cholesterol through tree nuts consumption. We also observed a similar significant reduction in TC. The first clinical trial in 1993 exploring the effects of walnuts in healthy men concluded that a walnuts diet can significantly decrease TC and LDL-C concentration (21). In addition, some well-controlled intervention trials with tree nuts also confirmed that tree nuts have cholesterol-lowering efficacy (48–52). When the population was limited to people with T2DM, our results showed that tree nuts and peanuts can significantly lower cholesterol concentration, which was consistent with the results of previous studies focused on healthy or hyperlipidemia patients. Nuts are rich in phytosterols, which may promote their TC-lowering effect (53).

Heterogeneity for TC may be explained by the duration of trials. We found a significantly greater reduction of TC concentration in studies whose duration was <12 weeks, it may be that the length of the intervention has a direct impact on the effect, and the longer intervention period and the shorter intervention period may have opposite outcomes. Also, given the relatively few studies for certain nut types, we did not observe significant differences in effects by nut type. In addition, we could not explore the possible source of heterogeneity for LDL-C. We attempted to explain heterogeneity by performing subgroup and sensitivity analyses, however, for the most part, heterogeneity remained. Also, no publication bias was detected for LDL-C, HDL-C, TC, and TG.

Peanuts and tree nuts, as a kind of nutritious food in daily life, are easy to obtain for subjects with type 2 diabetes. The management of blood lipids in patients with type 2 diabetic is also as important as blood sugar control and needs to be taken seriously. Our meta-analysis results revealed that daily intake of peanuts and tree nuts may help control TC and TGs. In addition, our results did not find that the intake of peanuts and tree nuts can lower HDL-C and LDL-C. Thus, peanut and tree nuts consumption can be considered as a healthy lifestyle to help manage the blood lipids in patients with type 2 diabetes.

This meta-analysis has several strengths. Our study examined the efficacy of tree nuts or peanuts intake in patients with type 2 diabetes. In addition, it was performed and reported based on current guidelines (25, 54, 55), and comprised an evaluation of results employing numerous sensitivity analyses, and investigation of the risk of bias using an updated assessment tool. There were potential limitations in our review. First, we did not prospectively register our systematic review and meta-analysis, so it may lack transparency and increase the potential for bias. Second, the majority of the trials used tree nuts or peanuts consumption as the dietary intervention at baseline. However, during the follow-up period, some participants may hide the fact that they have changed their dietary habits. Third, although random- or fixed-effects model selection based on the *I*^2^ metrics is commonly performed, preferably the model selection should be a priority. Whether the results from present studies are expected to produce one similar result across studies is unknown in advance although we have conducted a sensitivity analysis, which could be a limitation in our study. Fourth, we could not explore the effects of nut type because of the relatively few studies for certain nut types.

## Conclusion

In summary, evidence from our pooled analyses indicated that daily peanuts and tree nuts consumption may significantly reduce TC and TG concentration in patients with type 2 diabetes. In addition, no obvious evidence of an effect of daily peanuts and tree nuts supplementation on LDL-C concentration and HDL-C concentration was found. These findings support daily peanuts and tree nut intake, as an important part of healthy lifestyles, could be recommended in the management of blood lipids in type 2 diabetics subjects. Also, several larger-scale, high-quality randomized controlled trials of certain nut types are needed to validate the findings.

## Data Availability Statement

The datasets presented in this study can be found in online repositories. The names of the repository/repositories and accession number(s) can be found in the article/Supplementary Material.

## Author Contributions

J-yX and J-hY: conceptualization. D-fX and HX: data curation and software. J-yX, D-fX, and HX: formal analysis. J-yX and CY: methodology. J-yX and G-jS: supervision and validation. HX and G-jS: visualization. J-yX: writing—original draft. CY and G-jS: writing—review and editing. All authors contributed to the article and approved the submitted version.

## Conflict of Interest

The authors declare that the research was conducted in the absence of any commercial or financial relationships that could be construed as a potential conflict of interest.

## Publisher's Note

All claims expressed in this article are solely those of the authors and do not necessarily represent those of their affiliated organizations, or those of the publisher, the editors and the reviewers. Any product that may be evaluated in this article, or claim that may be made by its manufacturer, is not guaranteed or endorsed by the publisher.
