# Supplementary material for: The Effects of Peanuts and Tree Nuts on Lipid Profile in Type 2 Diabetic Patients: A Systematic Review and Meta-Analysis of Randomized, Controlled-Feeding Clinical Studies
Source: Front Nutr. 2021 Dec 1;8:765571. doi: 10.3389/fnut.2021.765571 (PMC8679310; doi:10.3389/fnut.2021.765571)
Supplement: Supplementary file 1 [file Data_Sheet_1.docx]

**List of supplementary data**

**Supplementary data 1:** PRISMA 2020 checklist

**Supplementary data 2:** Detailed search strategy

**Supplementary data 3:** Inclusion and exclusion criteria of relevant studies

**Supplementary data 4:** Sensitivity analyses using correlation coefficient of 0.25, 0.5, and 0.75 for cross-over studies

**Supplementary data 5:** Sensitivity analyses using the random-effects model with Hartung-Knapp-Sidik-Jonkman adjustment

**Supplementary data 6:** Results of ‘leave-one-out’ sensitivity analyses

**Supplementary data 7:** Summary Results of Subgroup Analyses from the Included Randomized Controlled-feeding Dietary Trials Evaluating effects of tree nuts and peanuts of patients with Type 2 diabetes

**Supplementary data 8:** GRADE assessment of the quality of the body of evidence

**Supplementary data 1:** PRISMA 2020 checklist

| **Section and Topic** | **Item #** | **Checklist item** | **Location where item is reported** |
| --- | --- | --- | --- |
| **TITLE** | | |  |
| Title | 1 | Identify the report as a systematic review. | Lines 1-3 |
| **ABSTRACT** | | |  |
| Abstract | 2 | See the PRISMA 2020 for Abstracts checklist. | Lines 13-39 |
| **INTRODUCTION** | | |  |
| Rationale | 3 | Describe the rationale for the review in the context of existing knowledge. | Lines 62-84 |
| Objectives | 4 | Provide an explicit statement of the objective(s) or question(s) the review addresses. | Lines 85-89 |
| **METHODS** | | |  |
| Eligibility criteria | 5 | Specify the inclusion and exclusion criteria for the review and how studies were grouped for the syntheses. | Lines 109-115 |
| Information sources | 6 | Specify all databases, registers, websites, organisations, reference lists and other sources searched or consulted to identify studies. Specify the date when each source was last searched or consulted. | Lines 94-108 |
| Search strategy | 7 | Present the full search strategies for all databases, registers and websites, including any filters and limits used. | Lines 90-105, Supplementary data 2 |
| Selection process | 8 | Specify the methods used to decide whether a study met the inclusion criteria of the review, including how many reviewers screened each record and each report retrieved, whether they worked independently, and if applicable, details of automation tools used in the process. | Lines 104-108 |
| Data collection process | 9 | Specify the methods used to collect data from reports, including how many reviewers collected data from each report, whether they worked independently, any processes for obtaining or confirming data from study investigators, and if applicable, details of automation tools used in the process. | Lines 117-118 |
| Data items | 10a | List and define all outcomes for which data were sought. Specify whether all results that were compatible with each outcome domain in each study were sought (e.g. for all measures, time points, analyses), and if not, the methods used to decide which results to collect. | Lines 118-120 |
|  | 10b | List and define all other variables for which data were sought (e.g. participant and intervention characteristics, funding sources). Describe any assumptions made about any missing or unclear information. | Lines 119-120 |
| Study risk of bias assessment | 11 | Specify the methods used to assess risk of bias in the included studies, including details of the tool(s) used, how many reviewers assessed each study and whether they worked independently, and if applicable, details of automation tools used in the process. | Lines 123-130 |
| Effect measures | 12 | Specify for each outcome the effect measure(s) (e.g. risk ratio, mean difference) used in the synthesis or presentation of results. | Lines 157-158 |
| Synthesis methods | 13a | Describe the processes used to decide which studies were eligible for each synthesis (e.g. tabulating the study intervention characteristics and comparing against the planned groups for each synthesis (item #5)). | Lines 148-149 |
|  | 13b | Describe any methods required to prepare the data for presentation or synthesis, such as handling of missing summary statistics, or data conversions. | Lines 149-153 |
|  | 13c | Describe any methods used to tabulate or visually display results of individual studies and syntheses. | Lines 153-155 |
|  | 13d | Describe any methods used to synthesize results and provide a rationale for the choice(s). If meta-analysis was performed, describe the model(s), method(s) to identify the presence and extent of statistical heterogeneity, and software package(s) used. | Lines 132-134, Lines 153-158 |
|  | 13e | Describe any methods used to explore possible causes of heterogeneity among study results (e.g. subgroup analysis, meta-regression). | Lines 158-166 |
|  | 13f | Describe any sensitivity analyses conducted to assess robustness of the synthesized results. | Lines 159-164 |
| Reporting bias assessment | 14 | Describe any methods used to assess risk of bias due to missing results in a synthesis (arising from reporting biases). | Lines 124-130 |
| Certainty assessment | 15 | Describe any methods used to assess certainty (or confidence) in the body of evidence for an outcome. | Lines 171-176 |
| **RESULTS** | | |  |
| Study selection | 16a | Describe the results of the search and selection process, from the number of records identified in the search to the number of studies included in the review, ideally using a flow diagram. | Lines 179-190 |
|  | 16b | Cite studies that might appear to meet the inclusion criteria, but which were excluded, and explain why they were excluded. | Lines 184-189 |
| Study characteristics | 17 | Cite each included study and present its characteristics. | Line 191-204 |
| Risk of bias in studies | 18 | Present assessments of risk of bias for each included study. | Line 205-208 |
| Results of individual studies | 19 | For all outcomes, present, for each study: (a) summary statistics for each group (where appropriate) and (b) an effect estimate and its precision (e.g. confidence/credible interval), ideally using structured tables or plots. | Lines 209-247 |
| Results of syntheses | 20a | For each synthesis, briefly summarise the characteristics and risk of bias among contributing studies. | Lines 206-208 |
|  | 20b | Present results of all statistical syntheses conducted. If meta-analysis was done, present for each the summary estimate and its precision (e.g. confidence/credible interval) and measures of statistical heterogeneity. If comparing groups, describe the direction of the effect. | Lines 209-247 |
|  | 20c | Present results of all investigations of possible causes of heterogeneity among study results. | Lines 249-256 |
|  | 20d | Present results of all sensitivity analyses conducted to assess the robustness of the synthesized results. | Lines 257-262 |
| Reporting biases | 21 | Present assessments of risk of bias due to missing results (arising from reporting biases) for each synthesis assessed. | Lines 205-208 |
| Certainty of evidence | 22 | Present assessments of certainty (or confidence) in the body of evidence for each outcome assessed. | Lines 278-283, Supplementary data 8 |
| **DISCUSSION** | | |  |
| Discussion | 23a | Provide a general interpretation of the results in the context of other evidence. | Lines 286-301 |
|  | 23b | Discuss any limitations of the evidence included in the review. | Lines 352-361 |
|  | 23c | Discuss any limitations of the review processes used. | Line 352 |
|  | 23d | Discuss implications of the results for practice, policy, and future research. | Lines 363-369 |
| **OTHER INFORMATION** | | |  |
| Registration and protocol | 24a | Provide registration information for the review, including register name and registration number, or state that the review was not registered. | none |
|  | 24b | Indicate where the review protocol can be accessed, or state that a protocol was not prepared. | none |
|  | 24c | Describe and explain any amendments to information provided at registration or in the protocol. | none |
| Support | 25 | Describe sources of financial or non-financial support for the review, and the role of the funders or sponsors in the review. | Line 384 |
| Competing interests | 26 | Declare any competing interests of review authors. | Line 370 |
| Availability of data, code and other materials | 27 | Report which of the following are publicly available and where they can be found: template data collection forms; data extracted from included studies; data used for all analyses; analytic code; any other materials used in the review. | Line 372-374 |

*From:*  Page MJ, McKenzie JE, Bossuyt PM, Boutron I, Hoffmann TC, Mulrow CD, et al. The PRISMA 2020 statement: an updated guideline for reporting systematic reviews. BMJ 2021;372:n71. doi: 10.1136/bmj.n71

**Supplementary data 2:** Detailed search strategy

**PubMed:**

| #1 Nuts or Tree nuts |
| --- |
| #2 pistachios |
| #3 pine nuts |
| #4 brazil nuts |
| #5 cashews |
| #6 hazelnuts |
| #7 almonds |
| #8 walnuts |
| #9 pecans |
| #10 macadamia nuts |
| #11 peanuts |
| #12 cholesterol, HDL or Cholesterol, LDL OR Triglycerides OR serum lipids OR triglyceride OR low density lipoprotein OR high density lipoprotein OR TG OR TC OR lipid profile OR serum lipid |
| #13 Type 2 diabetes OR diabetes mellitus, type II OR type 2 diabetes mellitus OR type 2 diabetes OR Diabetes, Type 2 |
| #14 randomized controlled trial OR randomized controlled trial OR controlled clinical trial OR clinical trial, randomized OR randomized, trial OR randomized OR intervention OR controlled trial OR random OR placebo |
| #15 1 or 2 or 3 or 4 or 5 or 6 or 7 or 8 or 9 or 10 or 11 |
| #16 #15 AND #12 AND #13 AND #14 |

**Embase:**

| #1 Nuts or Tree nuts |
| --- |
| #2 pistachios |
| #3 pine nuts |
| #4 brazil nuts |
| #5 cashews |
| #6 hazelnuts |
| #7 almonds |
| #8 walnuts |
| #9 pecans |
| #10 macadamia nuts |
| #11 peanuts |
| #12 cholesterol, HDL or Cholesterol, LDL OR Triglycerides OR serum lipids OR triglyceride OR low density lipoprotein OR high density lipoprotein OR TG OR TC OR lipid profile OR serum lipid |
| #13 Type 2 diabetes OR diabetes mellitus, type II OR type 2 diabetes mellitus OR type 2 diabetes OR Diabetes, Type 2 |
| #14 randomized controlled trial OR randomized controlled trial OR controlled clinical trial OR clinical trial, randomized OR randomized, trial OR randomized OR intervention OR controlled trial OR random OR placebo |
| #15 1 or 2 or 3 or 4 or 5 or 6 or 7 or 8 or 9 or 10 or 11 |
| #16 #15 AND #12 AND #13 AND #14 |

**Cochrane Library:**

| #1 Nuts or Tree nuts |
| --- |
| #2 pistachios |
| #3 pine nuts |
| #4 brazil nuts |
| #5 cashews |
| #6 hazelnuts |
| #7 almonds |
| #8 walnuts |
| #9 pecans |
| #10 macadamia nuts |
| #11 peanuts |
| #12 cholesterol, HDL or Cholesterol, LDL OR Triglycerides OR serum lipids OR triglyceride OR low density lipoprotein OR high density lipoprotein OR TG OR TC OR lipid profile OR serum lipid |
| #13 Type 2 diabetes OR diabetes mellitus, type II OR type 2 diabetes mellitus OR type 2 diabetes OR Diabetes, Type 2 |
| #14 randomized controlled trial OR randomized controlled trial OR controlled clinical trial OR clinical trial, randomized OR randomized, trial OR randomized OR intervention OR controlled trial OR random OR placebo |
| #15 1 or 2 or 3 or 4 or 5 or 6 or 7 or 8 or 9 or 10 or 11 |
| #16 #15 AND #12 AND #13 AND #14 |

**Web of science:**

| #1 Nuts or Tree nuts |
| --- |
| #2 pistachios |
| #3 pine nuts |
| #4 brazil nuts |
| #5 cashews |
| #6 hazelnuts |
| #7 almonds |
| #8 walnuts |
| #9 pecans |
| #10 macadamia nuts |
| #11 peanuts |
| #12 cholesterol, HDL or Cholesterol, LDL OR Triglycerides OR serum lipids OR triglyceride OR low density lipoprotein OR high density lipoprotein OR TG OR TC OR lipid profile OR serum lipid |
| #13 Type 2 diabetes OR diabetes mellitus, type II OR type 2 diabetes mellitus OR type 2 diabetes OR Diabetes, Type 2 |
| #14 randomized controlled trial OR randomized controlled trial OR controlled clinical trial OR clinical trial, randomized OR randomized, trial OR randomized OR intervention OR controlled trial OR random OR placebo |
| #15 1 or 2 or 3 or 4 or 5 or 6 or 7 or 8 or 9 or 10 or 11 |
| #16 #15 AND #12 AND #13 AND #14 |

**Scopus:**

| #1 Nuts or Tree nuts |
| --- |
| #2 pistachios |
| #3 pine nuts |
| #4 brazil nuts |
| #5 cashews |
| #6 hazelnuts |
| #7 almonds |
| #8 walnuts |
| #9 pecans |
| #10 macadamia nuts |
| #11 peanuts |
| #12 cholesterol, HDL or Cholesterol, LDL OR Triglycerides OR serum lipids OR triglyceride OR low density lipoprotein OR high density lipoprotein OR TG OR TC OR lipid profile OR serum lipid |
| #13 Type 2 diabetes OR diabetes mellitus, type II OR type 2 diabetes mellitus OR type 2 diabetes OR Diabetes, Type 2 |
| #14 randomized controlled trial OR randomized controlled trial OR controlled clinical trial OR clinical trial, randomized OR randomized, trial OR randomized OR intervention OR controlled trial OR random OR placebo |
| #15 1 or 2 or 3 or 4 or 5 or 6 or 7 or 8 or 9 or 10 or 11 |
| #16 #15 AND #12 AND #13 AND #14 |

**Supplementary data 3:** Inclusion and exclusion criteria of relevant studies

| **First author, year (ref)** | **Inclusion criteria** | **Exclusion criteria** |
| --- | --- | --- |
| Lovejoy et al, 2002(1) | Subjects were 30–65 year of age with a BMI of 20–40; Type 2 diabetes previously diagnosed by a physician or the subjects had a fasting serum glucose concentration > 7.8 mmol/L (140 mg/dL); have moderately good glucose control (fasting glucose <11.1 mmol/L, or <200 mg/dL). | Subjects taking insulin; subjects taking medications to lower cholesterol; those with clinically significant nephropathy, neuropathy, or cardiovascular disease; LDL-cholesterol concentrations were >5.2 mmol/L (200 mg/dL), fasting triacylglycerol concentrations were >7.8 mmol/L (300 mg/dL), or HDL-cholesterol concentrations were <0.65 mmol/L (25 mg/dL). |
| TAPSELL et al, 2004(2) | Subjects aged 35–75 years; diagnosed with type 2 diabetes for at least 1 year, and generally well. | Insulin therapy (or with HbA1c ＞9%), BMI ＞35 kg/m2 with major debilitating illness, known food allergies or food habits inhibiting the study, illiteracy, and inadequate conversational English. |
| Tapsell et al, 2009(3) | Subjects aged 35–75 years; previously diagnosed with T2DM, not insulin-treated, body mass index (BMI)＞25 and＜32 kg/m2; waist circumference ＞102 for men and＞94cm for women; and generally well. | Major illnesses; food allergies or inhibitory habits, illiteracy and/or inadequate English. |
| MA et al, 2010(4) | A clinical diagnosis of type 2 diabetes for at least 1 year but no more than 5 years; nonsmokers aged 30–75 years; had serum glucose levels and medication doses that had been stable for 3 months, and were not currently receiving insulin therapy. | Current use of vasoactive medications or supplements; current eating disorder, known athero-sclerotic vascular disease, sleep apnea, pregnancy, restricted diet, allergy to walnuts or other nuts, and use of lipidlowering or antihypertensive medications unless stable condition with medication for at least 3 months, and willingness to refrain from taking medication for 12 h before assessment. |
| Li et al, 2011(5) | T2DM patients previously diagnosed by a physician; (1) serum cholesterol greater than 200 mg/dL or triglycerol greater than 150 mg/dL, free of dietary restrictions/food allergies; (2) not receiving insulin therapy; (3) not using medications or supplementations known to alter lipid metabolism; (4) stable blood lipid and sugar levels within 3 months before study; (5) no clinical history of cardiovascular, hepatic, gastrointestinal, or renal disease; (6) no alcoholism; and (7) no recent history of smoking. | Not meet all of the eligibility criteria. |

(*continued*)

**Supplementary data 3:** Inclusion and exclusion criteria of relevant studies **(*continued*)**

| **First author, year (ref)** | **Inclusion criteria**  **Inclusion criteria** | **Exclusion criteria** |
| --- | --- | --- |
| Jenkins et al, 2018(6) | Men or postmenopausal women with type 2 diabetes who were taking antidiabetic agents other than acarbose, with medications stable for the previous 3 months and who had HbA1c values at screening between 6.5 and 8.0%. | Clinically significant cardiovascular, renal, or liver disease (alanine aminotransferase more than three times the upper limit of normal) or a history of cancer. |
| Cohen et al, 2011(7) | Adults with diagnosed T2D diagnosed by a physician at least 1 year before the start of the trials; had not been prescribed insulin; reported active disease conditions aside T2D. | Non-compliance with inclusion criteria |
| Darvish Damavandi et al, 2013(8) | Previously diagnosed with type 2 diabetes based on FBS >126 mg/dl or 2‑h blood sugar ≥200mg/dl, serum TGs <400 mg/dl, body mass index (BMI) ≤35 kg/m2, Hemoglobin‑A1C (HbA1C) <9%, serum LDL‑C <200 mg/dl, and blood pressure ≤160/90 mmHg | Any known allergies to nuts, insulin therapy, cigarette smoking, history of stroke, heart disease or thyroid disorders, diabetic nephropathy or retinopathy, or following vegetarian or weight‑loss diets up to 2 months before the study；Those patients who had consumed nuts more than 2‑times/week and changed their medications (type or dosage) up to 2 months before the study. |
| Sweazea et al, 2014(9) | Adults, 25–75 years of age, diagnosed by a physician with T2D for at least 6 months, | A history of peanut and/or tree nut allergy, insulin use, hemoglobin A1c (HbA1c) <6.5% or >9.0%, dietary intake of >12% monounsaturated fatty acids, active disease states (other than diabetes), anticipated change in diet or physical activity levels, and pregnancy or lactation. |
| Wien et al, 2014(10) | Adults with a medical diagnosis of T2D for at least 6 months and HbA1c less than 9.0%. | Individuals less than 20 years of age, that smoked, had nut allergies or a history of irritable bowel disease or diverticulitis that could be exacerbated by daily peanut intake, Patients with liver disease, renal disease and/or severe dyslipidemia (TG >4.52 mmol/l or TC >7.77 mmol/l). Use of long-acting insulin and statins were permitted if the dose was stable for at least 3 months. |

(*continued*)

**Supplementary data 3:** Inclusion and exclusion criteria of relevant studies **(*continued*)**

| **First author, year (ref)** | **Inclusion criteria**  **Inclusion criteria** | **Exclusion criteria** |
| --- | --- | --- |
| Sauder et al, 2015(11) | A self-reported diagnosis of type 2 diabetes; aged 30–75 years (women had to be post-menopausal), and have a body mass index (BMI) of 18.5-45.0 kg/m2. | Insulin use, self-reported history of chronic disease other than type 2 diabetes, history of bariatric surgery, major surgery in the prior 6 months, nut or latex allergies, and use of tobacco, daily aspirin, anti-inflammatory medications, oral steroids, hormone replacement therapy, or anti-hypertensive medication. blood pressure ≥160/100 mmHg, abnormal electrocardiogram, fasting triglycerides ≥5.65 mmol/L, or HbA1c ≥7.4%. |
| Chen et al, 2017(12) | Patients previously diagnosed with T2DM aged between 40 and 70 years; BMI: 24 to 35 kg/m2, HbA1c: 6.5–10%, and regular use of prescribed oral ghypoglycemic, agents biguanide, DPP-4 inhibitor, and α-glucosidase inhibitor. | Regular use of insulin, oral steroids or anti-inflammatory agents, ≥5% body weight change in the last 6 months, diagnosed CVD, stroke, gastrointestinal diseases, inflammatory bowel disease, chronic kidney disease, hepatobiliary disease, renovascular disease, endocrine diseases, hyperuricemia, autoimmune diseases, active treatment for cancer of any type ≤1year, poor hypertension control, known allergies to nuts of any kind, frequent nut consumption (≥3 oz./week), regular use of any dietary supplements or homeopathic remedies, daily ethanol intake of ≥2 drinks and smoking. |
| Mohan et al, 2018(13) | Subjects aged 30–65 years; physician-diagnosed T2DM; duration of T2DM<10 years, and currently receiving oral hypoglycemic drugs. | Cashew nut allergy, currently receiving insulin therapy, glycated hemoglobin (HbA1c) >10%, LDL cholesterol >190mg/dL, total cholesterol >240mg/dL, TGs >300 mg/dL, and any known diabetes complications. |
| Sedaghat et al, 2019(14) | Type 2 diabetics are age above 30, FBS<250, A1c <8.5Hb, BMI<35kg/m2. | Failures of the liver, kidney, and heart, insulin, allergy to soy consumption and immune suppressor drugs, alcohol, and cigarettes |

**Supplementary data 4:** Sensitivity analyses using correlation coefficient of 0.25, 0.5, and 0.75 for cross-over studies

**Table S1:** sensitivity analyses using varying correlation coefficients for cross-over studies, LDL-C (mmol/L)

| Outcome | Weighted mean difference (95% CI) | Inconsistency (I^2^) |
| --- | --- | --- |
| Paired analysis (original analysis) | -0.06 (-0.19, 0.07), p=0.370 | 63.3% |
| Correlation coefficient: 0.25 | -0.11 (-0.25, 0.030), p=0.111 | 50.3% |
| Correlation coefficient: 0.5 | -0.11 (-0.25, 0.030), p=0.117 | 53% |
| Correlation coefficient: 0.75 | -0.11 (-0.24, 0.030), p=0.112 | 58.7% |

**Table S2:** sensitivity analyses using varying correlation coefficients for cross-over studies, HDL-C (mmol/L)

| Outcome | Weighted mean difference (95% CI) | Inconsistency (I^2^) |
| --- | --- | --- |
| Paired analysis (original analysis) | -0.01 (-0.06, 0.05), p=0.833 | 59.2% |
| Correlation coefficient: 0.25 | 0.00 (-0.04, 0.04), p=0.946 | 43.9% |
| Correlation coefficient: 0.5 | 0.01 (-0.01, 0.04), p=0.400 | 44% |
| Correlation coefficient: 0.75 | 0.01 (-0.03, 0.05), p=0.683 | 44.9% |

**Table S3:** sensitivity analyses using varying correlation coefficients for cross-over studies, Total cholesterol (mmol/L)

| Outcome | Weighted mean difference (95% CI) | Inconsistency (I^2^) |
| --- | --- | --- |
| Paired analysis (original analysis) | -0.18 (-0.15, -0.003), p=0.042 | 86.8% |
| Correlation coefficient: 0.25 | -0.14 (-0.27, -0.001), p=0.048 | 46.4% |
| Correlation coefficient: 0.5 | -0.14 (-0.26, -0.02), p=0.024 | 45% |
| Correlation coefficient: 0.75 | -0.12 (-0.24, 0.003), p=0.056 | 55% |

**Table S4:** sensitivity analyses using varying correlation coefficients for cross-over studies, Triglyceride (mmol/L)

| Outcome | Weighted mean difference (95% CI) | Inconsistency (I^2^) |
| --- | --- | --- |
| Paired analysis (original analysis) | -0.11 (-0.18, -0.040), p=0.002 | 25% |
| Correlation coefficient: 0.25 | -0.10 (-0.18, -0.02), p=0.012 | 0% |
| Correlation coefficient: 0.5 | -0.01 (-0.17, -0.02), p=0.010 | 0% |
| Correlation coefficient: 0.75 | -0.11 (-0.18, -0.03), p=0.004 | 0% |

**Supplementary data 5:** Sensitivity analyses using the random-effects model with Hartung-Knapp-Sidik-Jonkman adjustment

| Outcome | DerSimonian and Laird  (original analysis)  Weighted mean difference (95% CI) | Hartung-Knapp-Sidik-Jonkman Weighted mean difference (95% CI) |
| --- | --- | --- |
| LDL-C (mmol/L) | -0.11 (-0.25, 0.03), p=0.121 | -0.11 (-0.26, 0.04), p=0.138 |
| HDL-C (mmol/L) | 0.01 (-0.01, 0.04), p=0.400 | 0.00 (-0.01, 0.04), p=0.400 |
| Total cholesterol (mmol/L) | -0.12 (-0.25, 0), p=0.058 | -0.12 (-0.27, 0.02), p=0.095 |
| Triglyceride (mmol/L) | -0.10 (-0.17, -0.02), p=0.010 | -0.010 (-0.17, -0.02), p=0.010 |

**Supplementary data 6:** Results of ‘leave-one-out’ sensitivity analyses

**Figure S1:** Estimates for effect of peanuts and tree nuts consumption on LDL-C (mmol/L) in type 2 diabetes if one study was omitted

**Figure S2:** Estimates for effect of peanuts and tree nuts consumption on HDL-C (mmol/L) in type 2 diabetes if one study was omitted

**Figure S3:** Estimates for effect of peanuts and tree nuts consumption on TC (mmol/L) in type 2 diabetes if one study was omitted

**Figure S4:** Estimates for effect of peanuts and tree nuts consumption on TG (mmol/L) in type 2 diabetes if one study was omitted

**Supplementary data 7:** Summary Results of Subgroup Analyses from the Included Randomized Controlled-feeding Dietary Trials Evaluating effects of tree nuts and peanuts of patients with Type 2 diabetes

| **Index** | **Subgroup** | **No. of Trials** | **Weighted mean difference** | | ***P*** | **I2(%)** | ***P* value of heterogeneity** |
| --- | --- | --- | --- | --- | --- | --- | --- |
|  |  |  | **mean** | ***95%CI*** |  |  |  |
| **LDL-C** | Overall | 11 | -0.11 | -0.25, 0.03 | 0.117 | 53 | 0.022 |
|  | Nut type |  |  |  |  |  |  |
|  | Almonds | 3 | -0.04 | -0.47, 0.39 | 0.864 | 57.1 | 0.094 |
|  | walnuts | 1 | -0.40 | -0.65, -0.15 | 0.001 | NA | NA |
|  | soy nut | 1 | -0.39 | -0.66, -0.12 | 0.004 | NA | NA |
|  | pistachio | 1 | -0.04 | -0.19, 0.11 | 0.593 | NA | NA |
|  | cashew | 1 | 0.11 | -0.08, 0.30 | 0.254 | NA | NA |
|  | peanuts | 1 | 0.00 | -0.43, 0.43 | 1.000 | NA | NA |
|  | hazelnuts | 1 | 0.07 | -0.35, 0.49 | 0.742 | NA | NA |
|  | mixed nuts | 2 | -0.14 | -0.40, 0.12 | 0.292 | 0 | 0.71 |
|  | Nut dose (g/d) |  |  |  |  |  |  |
|  | ≥45g | 6 | -0.23 | -0.41, -0.06 | 0.008 | 53.7 | 0.055 |
|  | ＜45g | 5 | 0.08 | -0.06, 0.22 | 0.284 | 0 | 0.780 |
|  | Mean age of participants |  |  |  |  |  |  |
|  | ＜55 years | 2 | -0.13 | -0.62, 0.36 | 0.160 | 88.9 | 0.003 |
|  | ＞=55 years | 9 | -0.12 | -0.25, 0.02 | 0.099 | 33.4 | 0.151 |
|  | Study design |  |  |  |  |  |  |
|  | Parallel-controlled | 7 | -0.06 | -0.23, 0.10 | 0.451 | 42.7 | 0.106 |
|  | crossover | 4 | -0.18 | -0.45, 0.08 | 0.181 | 68.9 | 0.022 |
|  | Duration of trials |  |  |  |  |  |  |
|  | ＜12 weeks | 4 | -0.20 | -0.43, 0.03 | 0.085 | 70.7 | 0.017 |
|  | ≥12 weeks | 7 | -0.01 | -0.16, 0.13 | 0.749 | 21.8 | 0.263 |
|  | Controlled diets |  |  |  |  |  |  |
|  | Low-fat control | 5 | -0.09 | -0.24, 0.05 | 0.201 | 13.1 | 0.330 |
|  | Diabetic diet | 3 | -0.07 | -0.43, 0.30 | 0.724 | 79.0 | 0.009 |
|  | Others | 3 | -0.15 | -0.47, 0.18 | 0.367 | 59.3 | 0.086 |
| **TC** | Overall | 16 | -0.14 | -0.26, -0.02 | 0.024 | 45 | 0.03 |
|  | Nut type |  |  |  |  |  |  |
|  | Almonds | 6 | -0.03 | -0.22，0.16 | 0.786 | 0 | 0.676 |
|  | walnuts | 3 | -0.35 | -0.85，0.16 | 0.176 | 70.2 | 0.035 |
|  | soy nut | 1 | -0.52 | -0.86，-0.18 | 0.003 | NA | NA |
|  | pistachio | 1 | -0.15 | -0.30，0.00 | 0.053 | NA | NA |
|  | cashew | 1 | 0.04 | -0.12，0.20 | 0.625 | NA | NA |
|  | peanuts | 1 | 0.18 | -0.22，0.58 | 0.376 | NA | NA |
|  | hazelnuts | 1 | -0.23 | -0.73，0.27 | 0.364 | NA | NA |
|  | mixed nuts | 2 | -0.22 | -0.47，0.03 | 0.084 | 0 | 0.437 |
|  | Nut dose (g/d) |  |  |  |  |  |  |
|  | ≥45g | 10 | -0.18 | -0.30, -0.05 | 0.007 | 29.1 | 0.177 |
|  | ＜45g | 6 | -0.09 | -0.35, 0.17 | 0.498 | 55.2 | 0.048 |
|  | Mean age of participants |  |  |  |  |  |  |
|  | ＜55 years | 6 | -0.09 | -0.29，0.11 | 0.356 | 48.3 | 0.085 |
|  | ＞=55 years | 10 | -0.17 | -0.33，-0.01 | 0.035 | 42.3 | 0.076 |
|  | Study design |  |  |  |  |  |  |
|  | Parallel-controlled | 9 | -0.14 | -0.35, 0.06 | 0.168 | 62.8 | 0.006 |
|  | crossover | 7 | -0.16 | -0.27，0.04 | 0.007 | 0 | 0.523 |
|  | Duration of trials |  |  |  |  |  |  |
|  | ＜12 weeks | 6 | -0.22 | -0.37，-0.08 | 0.002 | 23.4 | 0.258 |
|  | ≥12 weeks | 10 | -0.07 | -0.24，0.10 | 0.422 | 44.9 | 0.060 |
|  | Controlled diets | 8 | -0.11 |  |  | 82.0 |  |
|  | Low-fat control | 8 | -0.18 | -0.33, -0.03 | 0.020 | 26.2 | 0.220 |
|  | High-fat control | 1 | -0.06 | -0.46, 0.34 | 0.771 | NA | NA |
|  | Diabetic diet | 3 | -0.11 | -0.51, 0.29 | 0.598 | 78.9 | 0.009 |
|  | others | 4 | -0.09 | -0.40, 0.22 | 0.570 | 52.7 | 0.096 |

**NA: not applicable**

**Supplementary data 8:** GRADE assessment of the quality of the body of evidence

| **Certainty assessment** | | | | | | | **№ of patients** | | **Effect** | | **Certainty** | **Importance** |
| --- | --- | --- | --- | --- | --- | --- | --- | --- | --- | --- | --- | --- |
| **№ of studies** | **Study design** | **Risk of bias** | **Inconsistency** | **Indirectness** | **Imprecision** | **Other considerations** | **walnut** | **control** | **Relative (95% CI)** | **Absolute (95% CI)** |  |  |
| **LDL-C** | | | | | | | | | | | | |
| 11 | randomised trials | serious ^a^ | serious ^b^ | not serious | not serious ^c^ | none | -/432 | -/446 | - | MD 0.11 lower (0.25 lower to 0.03 higher) | ⨁⨁◯◯ LOW | IMPORTANT |
| **HDL-C** | | | | | | | | | | | | |
| 10 | randomised trials | not serious | serious ^d^ | not serious | serious ^e^ | none | -/426 | -/439 | - | MD 0.01 (0.01 lower to 0.04 higher) | ⨁⨁◯◯ LOW | IMPORTANT |
| **TC** | | | | | | | | | | | | |
| 16 | randomised trials | not serious | serious ^f^ | not serious | not serious ^g^ | none | -/652 | -/668 | - | MD 0.14 lower (0.26 lower to 0.02 lower) | ⨁⨁⨁◯ MODERATE | IMPORTANT |
| **TG** | | | | | | | | | | | | |
| 13 | randomised trials | not serious | serious ^h^ | not serious | not serious^i^ | none | -/503 | -/520 | - | MD 0.10 lower (0.17 lower to 0.02 lower) | ⨁⨁⨁◯ MODERATE | IMPORTANT |

**CI:** Confidence interval

1. Four of the eleven studies had at least one item rated as high risk. In addition, there are some studies that have an item rated as high risk.
2. The direction of the effect size of the forest map in each study is inconsistent, and the degree of overlap is obviously small.
3. Sample size in review exceeds estimated Optimal Information Size. 95% confidence intervals in analysis do not cross appreciable harm/benefit and no effect. As a result, this outcome was not downgraded for imprecision.
4. The direction of the effect size of the forest maps in each study is inconsistent, and the degree of overlap is small.
5. Sample size in review are lower than estimated Optimal Information Size.
6. The direction of the effect size of the forest maps in each study is inconsistent.
7. Sample size in review exceeds estimated Optimal Information Size. 95% confidence intervals in analysis do not cross appreciable harm/benefit and no effect. As a result, this outcome was not downgraded for imprecision.
8. The direction of the effect size of the forest map in each study is inconsistent.
9. Sample size in review exceeds estimated Optimal Information Size. So this outcome was not downgraded for imprecision.
